# The Importance of Stochastic Effects for Explaining Entrainment in the Zebrafish Circadian Clock

**DOI:** 10.1155/2015/254979

**Published:** 2015-11-16

**Authors:** Raphaela Heussen, David Whitmore

**Affiliations:** ^1^CoMPLEX, UCL, Physics Building, Gower Place, London WC1E 6BT, UK; ^2^Department of Cell and Developmental Biology, UCL, Gower Street, London WC1E 6BT, UK

## Abstract

The circadian clock plays a pivotal role in modulating physiological processes and has been implicated, either directly or indirectly, in a range of pathological states including cancer. Here we investigate how the circadian clock is entrained by external cues such as light. Working with zebrafish cell lines and combining light pulse experiments with simulation efforts focused on the role of synchronization effects, we find that even very modest doses of light exposure are sufficient to trigger some entrainment, whereby a higher light intensity or duration correlates with strength of the circadian signal. Moreover, we observe in the simulations that stochastic effects may be considered an essential feature of the circadian clock in order to explain the circadian signal decay in prolonged darkness, as well as light initiated resynchronization as a strong component of entrainment.

## 1. Introduction

In evolution's continuous battle for the survival of the fittest, the ability to anticipate recurring environmental fluctuations has emerged as a powerful tool and immense selective advantage, allowing organisms to tailor their behaviour and biological processes to expected future opportunities and challenges. It may not be surprising that most living entities, from human [1] beings to cyanobacteria [2], make use of daily time-keeping mechanisms, also known as circadian clocks. These systems, however, are generally much more multifaceted than simple hour clocks, featuring, for example, the ability to adjust to different day light spans (photoperiods) and in doing so can even double as a useful seasonal timer.

Traditionally, the importance of time keeping is often considered on a behavioural level, for example, looking to the way different higher organisms would structure their daily or seasonal behaviour in line with an inner “sense of time”: flowers synching their blooming periods to day light or, more rarely, night time hours, nocturnal rodents sensing when to return to the safety of their burrows in time before dawn, or migratory birds punctually embarking on their yearly journeys across the globe. Many of us are also very familiar with the experience of waking up just a few minutes before the alarm clock, or having a good sense of when our usual meal times come around. It is also on this level that researches started looking at the circadian clock intently from a therapeutic angle, for example, in the context of chronic sleeping disorders, or the jet lag evoked by modern means of travel. Indeed, intercontinental flights that may expose us to radically opposed time zones in a matter of hours have demonstrated to us only too well the general flexibility, but also significant delay, with which our circadian rhythm adjusts to such radical perturbations.

As fascinating as the interactions of our subconscious and rational routines with our inner time keeper may be, it is also well worth remembering that there exists another, far deeper level to circadian rhythms. In fact, in many organisms the functioning, regulation, and adaptation of circadian time is effected at the cellular level, including of course all circadian clocks in unicellular organisms; but there are also more complex organisms and even vertebrates, such as zebrafish, where no central pacemaker has been identified and instead many cells and tissues contain autonomous circadian clocks [3, 4]. In many other species, a central circadian pacemaker has been identified in discrete regions in or close to the brain, such as the optic lobes of* Drosophila* or the suprachiasmatic nuclei (SCN) in the hypothalamus of mammals [5], but nevertheless the circadian signal remains closely integrated with cellular processes. Moreover, in human beings, for instance, recent research has made it clear that there exists not only one centrally controlled circadian clock, but additionally self-sustained oscillations in several tissues throughout the body [6]. These are referred to as peripheral oscillators and appear to control local rhythmic events [7].

These different layers of circadian regulation are speculated to play a vital part in controlling the molecular processes, communication, and life cycles of individual cells, and it is known, for example, that the critical process of mitosis, or cell division, is strictly timed around certain key checkpoints, such as the transition from G2 to M and G1 to S and the so-called metaphase checkpoint [8]. One rationale for this observed behaviour may be to minimize exposure of vulnerable DNA states to the destructive effects of UV radiation in the form of sun light and to coordinate the effects of damage-repair functions. Observations such as the preceding one are of course also acutely relevant for developing novel diagnostic and therapeutic approaches. Not only could understanding the exact role of the circadian clock in timing and modulating critical cellular processes offer insights into the disrupted states of cancer cells, but moreover this knowledge may also readily and significantly boost the outcome of therapeutic procedures. For example, in several models it was shown how, by simply varying the timing of the administration of chemotherapy, outcomes were significantly improved [9, 10]. Even this insight, however, is likely only the proverbial tip of the iceberg, as recent research also reveals implications of the circadian clock in a whole range of pathological states. This breadth may include cases, where a compromised state of the molecular basis of the circadian clock directly constitutes or contributes to the health problem, others in which the circadian clock may be indirectly affected, or those cases in which its cyclical effects on physiological processes may simply be leveraged to modulate treatment options.

One basic research area of particular note is the way in which external stimuli may interact with and adjust our inner time keeper. While even species living in constant darkness are known to possess circadian clocks, and an entire variety of potential environmental cues has been identified including feeding and tidal rhythms, the most prominent and important regulator remains exposure to sun light. Even in the absence of light input, the circadian clock follows an oscillating rhythm with a period usually close to 24 hours, which is termed the free running period and is specific to each species. In nature, however, the clock is habitually entrained by light input to the daily rhythm of exactly 24 hours. In this context, it is very interesting to note that in many species the overall signal strength of the circadian clock may also readily degrade in the absence of normal light/dark cycles. However, it has been demonstrated that individual oscillators continue functioning but will increasingly desynchronize over time [11]. This suggests, in turn, that the major way in which light entrains the circadian clock may only become apparent when considering synchronization effects at the cell population level.

This paper reports efforts to elucidate these complex interactions by investigating in particular sensitivity to synchronizing cues. A dual approach was adopted, in which a mathematical model of the zebrafish circadian clock key molecular components and their respective interactions was constructed and extended to capture the dynamics of natural desynchronization over time and resynchronization under the influence of light. Key behaviours from this stimulatory framework are compared and contrasted with results obtained from laboratory experiments, in which the effect of light pulses on desynchronized populations of zebra fish cells was measured.

## 2. Materials and Methods

### 2.1. The Zebrafish Circadian Clock

Zebrafish are not only recognized as an important vertebrate model species in general, but also an especially interesting candidate in the context of studying the circadian clock and light entrainment in particular [12]. One of the main reasons lies in the fact that they exhibit several similarities to mammals in the circadian clock makeup, but no central circadian pacemaker has been found in zebrafish, with timekeeping seemingly effected at a cellular level. This makes it possible to work with populations of zebrafish cell lines while limiting distortions due to interference due to centralized coupling, and so forth, as would be the case in many other vertebrates. Furthermore, individual cells are known to be very light sensitive and possess a direct light entrainment pathway including photopigments [13–15], allowing for a strong entrainment effect due to light exposure. Moreover, recent studies also point to many other aspects of cell biology being influenced by light-induced gene expression in zebrafish [16].

**Figure 1:**
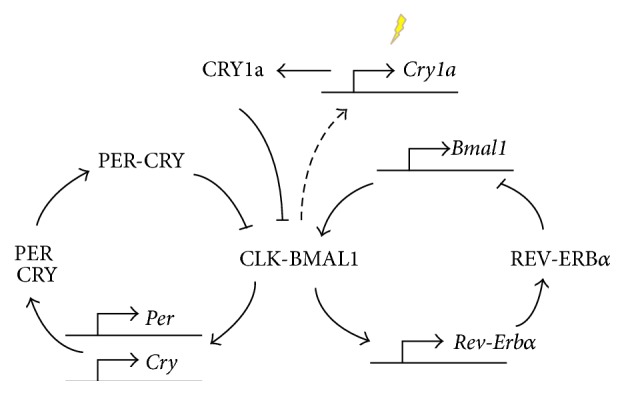
Core components of the circadian clock in zebrafish.

Looking at biological oscillators in general, negative feedback is essential, which ensures a network is carried back to its starting point, while a sufficient delay ensures that reactions do not settle on a stable steady state. It has been found that oscillations are impossible in a two-component negative feedback loop but require at least three components [17], and accordingly molecular feedback loops based on various clock genes have been identified. There is now also affirmation of nontranscriptional, posttranslational mechanisms, such as protein phosphorylation. Identified zebrafish clock genes include* Clock*,* Bmal*,* period*, and* cryptochrome* genes [12]. It should also be noted that one of the characteristic features of the zebrafish clock is the presence of extra copies of the key clock genes. The core clock components (see Figure 1) constitute an autoregulatory feedback loop, with Clock and Bmal1 heterodimerizing and activating transcription of* period* (*Per*) [18] and* cryptochrome* (*Cry*) genes, which in turn inhibit Clock/Bmal1. In addition, it was shown that Cry1a is upregulated by light and may directly interact with specific regions of Clock (PAS B) and Bmal1 (bHLH, PAS B, and C-terminal domains), blocking their ability to form an active dimer and initiate downstream transcriptional activation [19]. There is also a stabilizing feedback loop, where Rev-Erb *α* and Rora are believed to direct rhythmic expression of the Clock and Bmal genes.

As noted above, in the zebrafish circadian clock entrainment occurs primarily in response to light. Exposure triggers photoreceptors, their coupled signalling pathways, and finally a set of clock genes, namely,* per2* and* cry1a*. The clock also shows varied sensitivity to resetting cues; that is, depending on the time of day, light causes phase advances and delays or has no effect, whereby this resetting efficiency also correlates with the level of* Cry1a* upregulation. It can often be very useful to document a variable reaction to the same stimuli at different times of the day by constructing a phase response curve (PRC), and in the case of zebrafish cell lines the PRC shows the largest shift at late subjective night, causing a 15-hour shift, while at the early subjective day there is almost no phase shift observed.

### 2.2. Implementing a Mathematical Model

A first iteration of a zebrafish clock model was based on two interlocked negative feedback loops. The first one consists of the ClockBmal heterodimer and* Per1*, while the second one features ClockBmal and* Cry1a* plus a light input into* Cry1a*. The equations for the five ODEs are as follows:

(1) c1am′=v1ClkBmalka+ClkBmal−kdmc1amkdg+c1am+light,cry1a′=k1∗c1am−kdcry1akdg+cry1a,ClkBmal′=k2kc1nkc1n+cry1an+k3kc2nkc2n+per1n−kdClkBmalkdg+ClkBmal,p1m′=v2ClkBmalkp+ClkBmal−kdmp1mkdg+p1m,per1′=k4∗p1m−kdper1kdg+per1.

The ClockBmal heterodimer is denoted by* ClkBmal*, Cry1a mRNA and protein are denoted by* c1am* and* cry1a*, respectively, and Per1 mRNA and protein are denoted by* p1m* and* per1*, respectively. Parameter values that were used in simulations are given in Table 1.

When solving this system numerically, it is found that stable oscillations are readily achieved for degradation rates that are Michaelian rather than linear decay rates and a Hill coefficient of 4 or higher for the repression of ClockBmal by Cry1a and Per1. Here, Michaelis-Menten kinetics describe conversion from substrate to product based on enzyme concentration via a reversible formation of an enzyme-substrate complex and, following on from this, the irreversible release of the product. The Hill function would alter the quality of the response from a normal hyperbolic response to a more sigmoidal curve and would represent a form of ultrasensitivity. This reaction type occurs frequently in signalling pathways and, due to evoking shifts on a much smaller range, is considered economical for the cell. Mathematically, more extensive cooperativity would be represented by a higher Hill coefficient.

### 2.3. Importance of Stochastic Effects

While all models are, by their very nature, bound to be simplifications of real world processes, it can oftentimes be a complex and demanding challenge to arrive at a suitable balance of ensuring a truthful representation and ample predictive power on one hand, versus ease of implementation and a sufficiently “speedy” resolution on the other. One aspect that has seen a lot of attention in this area in recent years is the fact that many biological processes are inherently stochastic in nature, with attributes that can move randomly between different states in state space. In other words, fluctuating amounts and uncertain interactions of substance molecules at the microscopic level give rise to noisy and random events, which regularly defy the deterministic modelling assumption, whereby a given initial state always leads to the same state at a specific time later.

It is also very noteworthy that the nature of this noisiness is not restricted to the extrinsic variety, but rather it is found that even clonal, that is, genetically identical, cells exhibit significant deviations from each other in RNA and protein levels [11]. Following on from this, it has become apparent what an important role falls on intrinsic noise [20], which cannot be controlled for and stems from related chance events during promoter/DNA binding events, mRNA transcription and degradation, translation, and protein-protein interactions. In order to make sense of this observation, it can be useful to remember that genes are only present in a few (e.g., one or two) copies and transcription factor molecules in the order of tens or hundreds.

Moreover, it is now understood that noise, rather than simply being an unavoidable nuisance, can even be exploited by organisms; for example, in bistable systems cells can select from two phenotypes even in uniform genetic and surrounding conditions to facilitate adaptation to fluctuating environments. In the case of the circadian clock, it was also found that in some instances of the global signal averaging to a nonoscillating flat level, single cells may still have functional oscillators, albeit with widely fluctuating peaks and thereby “cancelling out” each others' signals [11]. In order to investigate the prominence of stochastic behaviour in the modelling of the zebrafish circadian clock, the simulation was extended to be solved numerically as a Stochastic Differential Equation (SDE) with a noise term driving randomly drifting substance concentrations.

The implemented SDEs were based on the ODE model described above, but the final concentrations at each time step were subjected to a noise term, here constant white noise on the basis of the Wiener process. The SDE model stimulations were implemented in Matlab using the “sde_euler” function. It should be noted that the Wiener process is very complex mathematically and practically impossible to differentiate, and consequently specific rules had to be devised to handle this kind of stochastic calculus, the two most widely used versions being Ito and Stratonovich stochastic calculus. Here, the Stratonovich type using Euler-Heun method is utilized.

### 2.4. Light Pulse Experiment

Following on from the theoretical simulations described above, a laboratory experiment investigating the synchronizing effect of exposing zebrafish cell line populations on a single light pulse, as well as possible thresholds to this synchronization in particular, was conducted utilizing a bioluminescence assay.

For these experiments, a* period1-luciferase* reporter cell line was used to monitor gene expression and progression of the circadian oscillator. The enzyme luciferase, synthesized when transcription is activated, interacts with the substrate luciferin, which can be added to the medium, to release light by the process of bioluminescence. This bioluminescence can then be systematically detected and measured as counts per seconds (CPS). While it is also theoretically possible to look at a single cell level, the experimental setup and bioluminescence detection are much more challenging, as a single cell produces relatively few photons [5]. Accordingly, most bioluminescence experiments utilize populations of different cell lines, each holding a specific clock reporter gene construct and thus allowing us to look at various transcriptional activities with high time resolution.

In this particular light pulse experiment, an existing* per1-luciferase* zebrafish cell line was used and the details of its creation can be reviewed [18]. The* per1-luciferase* cells were plated in quadruplicate wells of a 96-well plate (with approximately 25 × 10^3^ cells per well) in media containing 0.5 mM beetle luciferin. For each light intensity, one separate plate was used, and plates were kept in a dark incubator for 5 days before data recording. Light pulses were performed at the time, intensity, and duration as indicated on the figures. Bioluminescence was monitored on a Packard TopCount NXT scintillation counter. For light pulses, the plates were taken out of the Packard scintillation counter and kept in a dark chamber until light pulsed at the desired intensity.

Finally, before quantifying the synchronization strength of light on asynchronous cell population, by such measures as amplitude decay or the amplitude just after the pulse, underlying trends are removed from the data sets using a Hilbert Transform. Such trends may occur in bioluminescence circadian rhythms in cultured cells for several reasons and hinder the quantitative analysis. Firstly, the response of cell cultures to different treatments is variable and may be influenced by unaccounted factors. Secondly, the rhythms of the cell cultures exhibit damping (i.e., variance nonstationarities). Thirdly, these rhythms often show unstable baseline shifting (i.e., mean nonstationarities) that changes from experiment to experiment, or even from sample to sample. The procedure to remove baseline drift that can mask the circadian rhythm is quite simple and involves subtracting a 24-hour moving average from the raw data.

## 3. Results and Discussion

**Figure 2:**
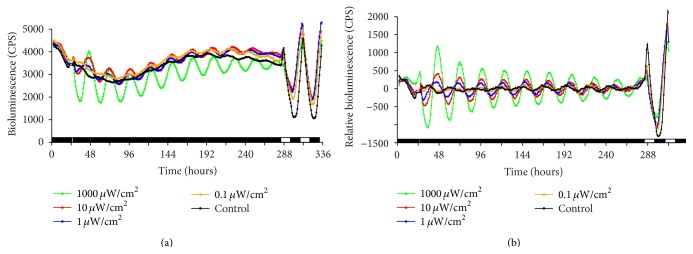
15-minute light pulse experiments at varying intensity. Bioluminescence trace of* Per1* reporter cell line. Cells were kept in the dark for 5 days before data recording. A 15-minute light pulse of varying strength as indicated was administered at about 24 hours (control, no light pulse). At the end of the experiment, cells were kept in LD for two days. This was done to confirm that cells were still healthy and responded to light as expected. (a) Raw data; (b) detrended data. The black and white boxes at the bottom of the graph indicate lights on (white) and lights off (black).

**Figure 3:**
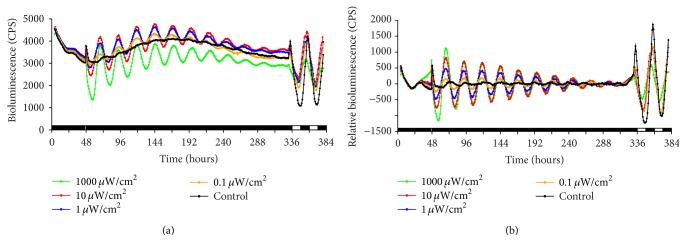
1-hour light pulse experiments at varying intensity. Bioluminescence trace of* Per1* reporter cell line. Cells were kept in the dark for 5 days before data recording. A 1-hour light pulse of varying strength as indicated was administered at about 48 hours (control, no light pulse). At the end of the experiment, cells were kept in LD for two days. This was done to confirm that cells were still healthy and responded to light as expected. (a) Raw data; (b) detrended data. The black and white boxes at the bottom of the graph indicate lights on (white) and lights off (black).

The experiment was carried out as described above with light pulse durations of 15 minutes and 1 hour and light intensities ranging from 0.1 to 1000 *μ*W cm^−2^, where a short duration and low intensities were chosen to determine what amount of light may be sufficient to cause an effect. The wavelength spectrum was 400–700 nm, and assuming a wavelength of 520 nm this range of irradiance corresponds to a photon flux of 0.0043 to 43 *μ*mol m^−2^ s^−1^. The bioluminescence traces for the 15-minute and 1-hour experiments can be seen in Figures 2 and 3, respectively.

**Figure 4:**
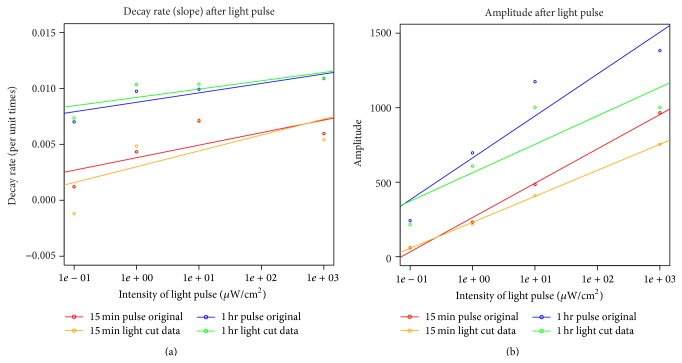
Decay rate of amplitude after exposure to light pulses of different duration (15 minutes or 1 hour) and light intensities. Decay rate and amplitude are shown for the different length of light pulse and intensities of light. Additionally, the first complete cycle of the original detrended data was ignored for the cut data set.

The traces were detrended using a 24-hour moving average and the amplitude after the light pulse and the decay rate were determined using a Hilbert Transform. The results of the decay rate and amplitude analysis can be seen in Figure 4.

These figures show that, with increasing length of the light pulse or intensity, population oscillation amplitude is increased. At the single cell level, zebrafish cells actually keep oscillating, even in darkness. The lack of oscillations on the population level can be explained by a desynchronous population. Light synchronized the population and only now the oscillations can be seen at the population level.

The decay rate seems to increase slightly with higher light intensity and longer light pulse. It might be the case that the more synchronized the population, and thus featuring a larger selection of individual oscillators forced into a common phase, the faster the synchronized state decays in the absence of the entraining signal, as individual oscillators with strong divergence move quickly away from this state. However, this aspect of the model proves difficult to explore and compare experimentally as single cell experiments are costly to set up, especially at the large scale required to verify individual variation effects. As expected due to a higher level of synchronization, the amplitude also increases with higher light intensity and longer light pulse.

Considering the fact that the number of photons stimulating the cell is proportional to light intensity times duration, it is interesting to note that 10 *μ*W cm^−2^ for 15 minutes has a lower initial amplitude than the 1 *μ*W cm^−2^ for 1 hour, implying that the cell does not simply take account of the number of photons. In order to check if the light pulses also had an immediate effect, the data points for the first full cycle were removed and the analysis was performed again, showing that interestingly the decay rate between the original and “cut” data is very similar; only the amplitude is smaller, as would be expected.

**Figure 5:**
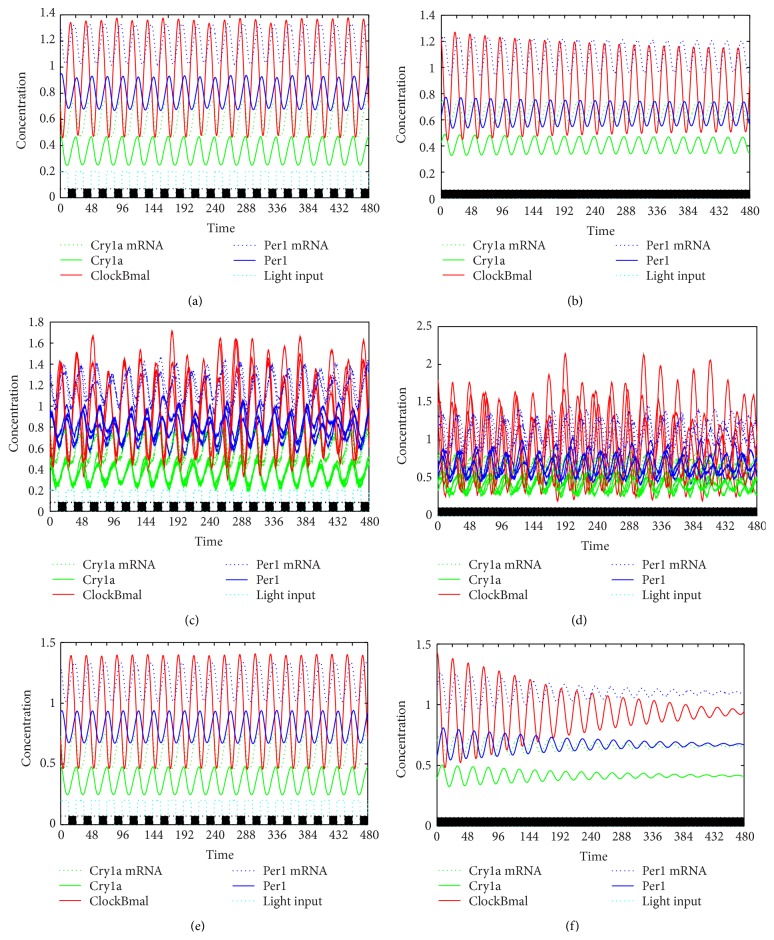
Stochastic versus deterministic simulations under different light regimes. The first row depicts deterministic oscillations under regular light dark cycles (LD, shown on the left) and constant darkness (DD, shown on the right), respectively. The second row shows individual oscillators that have been subjected to stochastic drift under the same light conditions as column one. The third row plots the average of 1000 stochastic oscillators. It can be seen that while there is very good match under LD conditions, only the stochastic property leads to signal dampening in DD. The black and white boxes at the bottom of the graph indicate lights on (white) and lights off (black). Cry1a mRNA is shown in green dotted line, Cry1a in green solid line, ClockBmal dimer in red line, Per1 mRNA in blue dotted line, and Per1 in blue solid line. Light blue dotted line shows light input/intensity.

**Figure 6:**
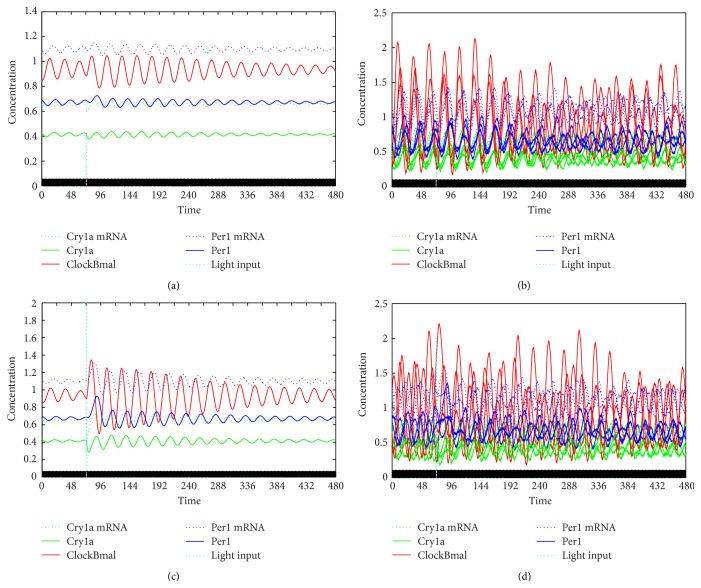
Stochastic simulations after light pulses. All traces show stochastic stimulations that were running freely in constant darkness before being subjected to a single light pulse at low (top) and high (bottom) intensities. The right column depicts five individual oscillators, while the left column shows the average of 1000. The results are largely in line with the experimental data. The black and white boxes at the bottom of the graph indicate lights on (white) and lights off (black). Cry1a mRNA is shown in green dotted line, Cry1a in green solid line, ClockBmal dimer in red line, Per1 mRNA in blue dotted line, and Per1 in blue solid line. Light blue dotted line shows light input/intensity.

Having obtained the experimental data presented above, we attempted to replicate in the model simulation the effect of a variable phase response, namely that light can either advance or delay the circadian rhythm, or have no effect depending on the specific timing of light pulses, thereby resetting the clocks in asynchronous populations to a common phase (see Figure 5 for a comparison of deterministic and stochastic simulations). The experimental setup was approximated in silico; that is, 1000 stochastic oscillators were desynchronized in the absence of light and subsequently exposed to light pulses of varying intensity. The resulting traces are found in Figure 6.

Comparing and contrasting the results obtained in bioluminescence assay experiments and corresponding model simulations, it appears that important behavioural aspects have been approximated by even a relatively basic model. Of course, some caution should be exercised interpreting these results. Although readings for each separate light pulse run were based on 4 individual wells and appear to correspond well over the range of different traces, the experimental design may still have been subject to distortions. In fact, since the very nature of the circadian clock's signal decay in zebrafish appears to be driven by stochastically drifting free running periods and resulting desynchronization of individual cellular oscillators, it is clear that this very randomness would also reveal itself in an experimental setup. Looking to the simulation runs, in turn, it can be noted on detailed inspection that, for instance, timing of peaks for individual substrates does not correspond to those reported in the literature (e.g.,* Cry1a* is reported to peak during daytime, but in the simulation peaks at the end of the night). This puzzling observation further highlights the complexities of the circadian clock, and the fact that the model at this stage captures well one set of behaviours, but not others.

One other result that emerges, however, is the fact that no hard lower or higher limit to the synchronization effect was detected at the range of light intensities employed. Rather it appears that there is a relatively constant relationship between the intensity of the light stimulation and resulting overall amplitude in the case of the 15-minute light pulses. For the 1-hour pulses, on the other hand, there appears to occur some levelling off at higher intensities, suggesting that the linear relationship no longer holds true and some kind of saturating effect could be inferred. It would be interesting to explore this further experimentally at additional intensities and pulse durations. It appears likely that amplitude shifts following light exposure are due to the resynchronization of asynchronous individual oscillators, and in this context, it could be argued that stronger pulses succeed in harmonizing the phases of individual oscillators more removed from the average. It is confirmed, however, that light pulses of only 15 minutes are generally sufficient to evoke a clear response from completely asynchronous cell populations.

## 4. Conclusion

In conclusion, the results of this study support the suggestion that synchronization between individual oscillators at the cellular level constitutes a major component of generating a stable circadian signal. In turn, external entrainment by light appears to align these oscillators with one another, while stochastic effects allow them to drift apart. This seemingly harmless observation actually contains important implications for the treatment of cancer and other ailments. Not only was the point made before that better understanding of the circadian clock could be leveraged here via its strong controlling influence on other cellular processes; rather, there is also more underlying realization, which in the age of evidence based medicine and its focus on clearly cut deterministic understanding of “doing A causes B” may otherwise be overlooked. Namely, that stochastic behaviour not only is a distraction but also may be a fundamental part of the functioning of basic physiological processes and their regulation. As such, it should be an important challenge for the years ahead to better understand and learn to work with this kind of inherent variability and also to understand how fundamental cell biology takes advantage of inherent stochastic noise.

## Figures and Tables

**Table 1:** 

*v*_1_	*v*_2_	*k*_*dm*_	*k*_deg⁡_	*k*_*d*_	*k*_1_	*k*_2_	*k*_3_	*k*_4_	*k*_*a*_	*k*_*p*_	*k*_cl1_	*k*_cl2_	Light	*n*
0.315	0.405	0.405	0.45	0.45	0.325	0.405	0.567	0.216	0.243	0.324	0.405	0.405	0.05	4
